# Supplementary material for: Amine Functionalization Leads to Enhanced Performance for Nickel- and Cobalt-Ferrite-Supported Palladium Catalysts in Nitrobenzene Hydrogenation
Source: Int J Mol Sci. 2023 Aug 28;24(17):13347. doi: 10.3390/ijms241713347 (PMC10487572; doi:10.3390/ijms241713347)
Supplement: Supplementary file 1 [file ijms-24-13347-s001.zip › ijms-2558206-supplementary.pdf]

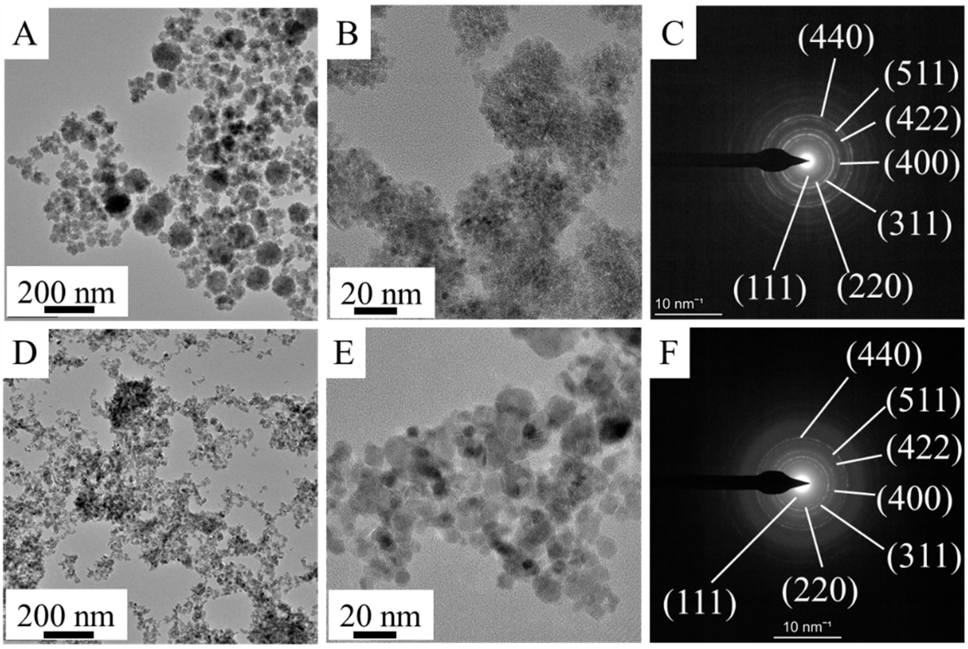

FigS1: HRTEM pictures and electron diffraction (SAED) of the amine-functionalized (A, B and C) and non-functionalized (D, E and F) $NiFe_2O_4$ catalyst support

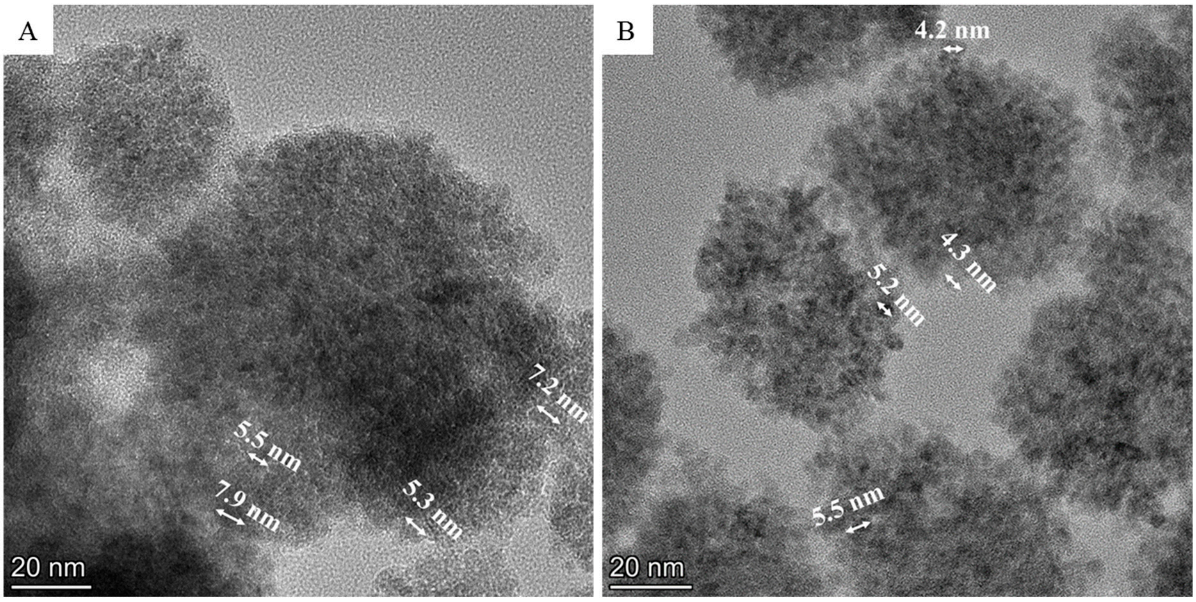

FigS2: HRTEM pictures of the $CoFe_2O_4$- NH_2 (A) and $NiFe_2O_4$-NH_2 (B) nanoparticles

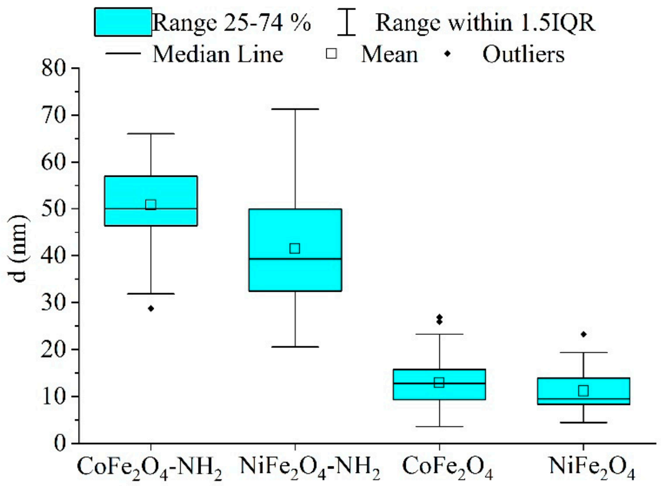

FigS3: Box-plot diagramms of the particle sizes of the ferrite catalyst supports

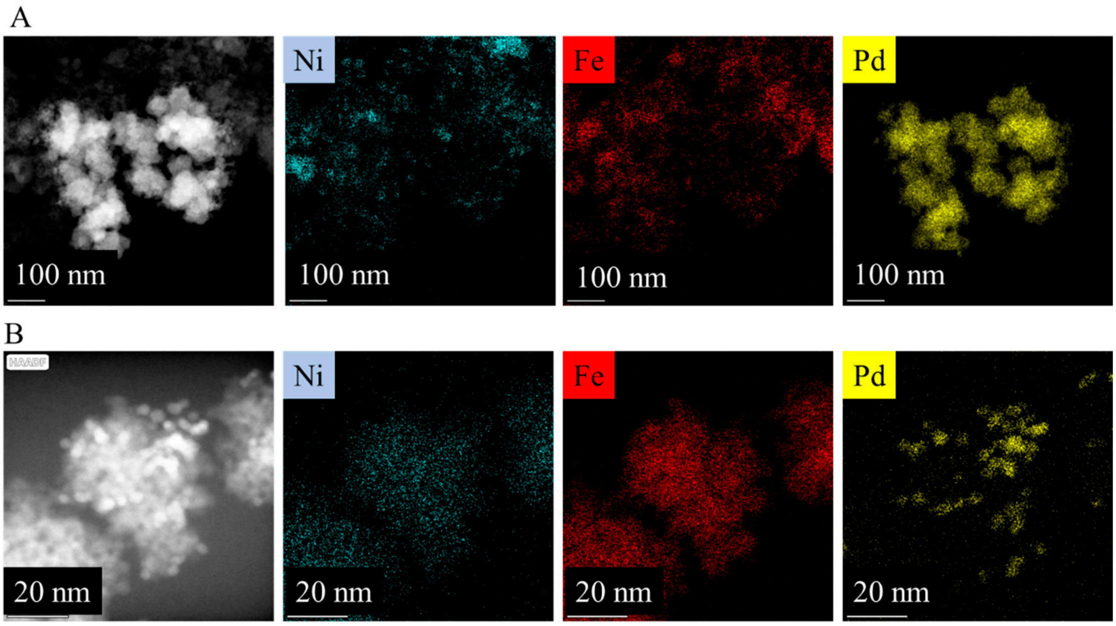

FigS4: Element mapping of the amine-functionalized (A) and the non-functionalized (B) Pd/$NiFe_2O_4$ catalysts

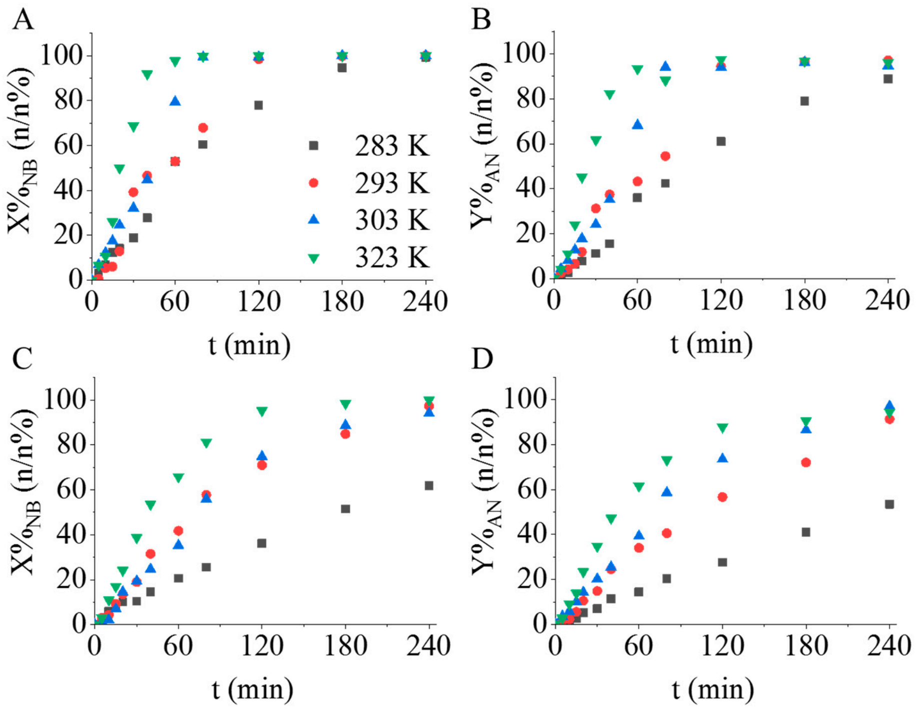

FigS5. Nitrobenzene conversions and aniline yields vs. time of hydrogenation at 283 K, 293 K 303 K and 323 K by using of Pd/$NiFe_2O_4$ (A, B) Pd/$NiFe_2O_4$-NH_2 (C, D) catalysts.

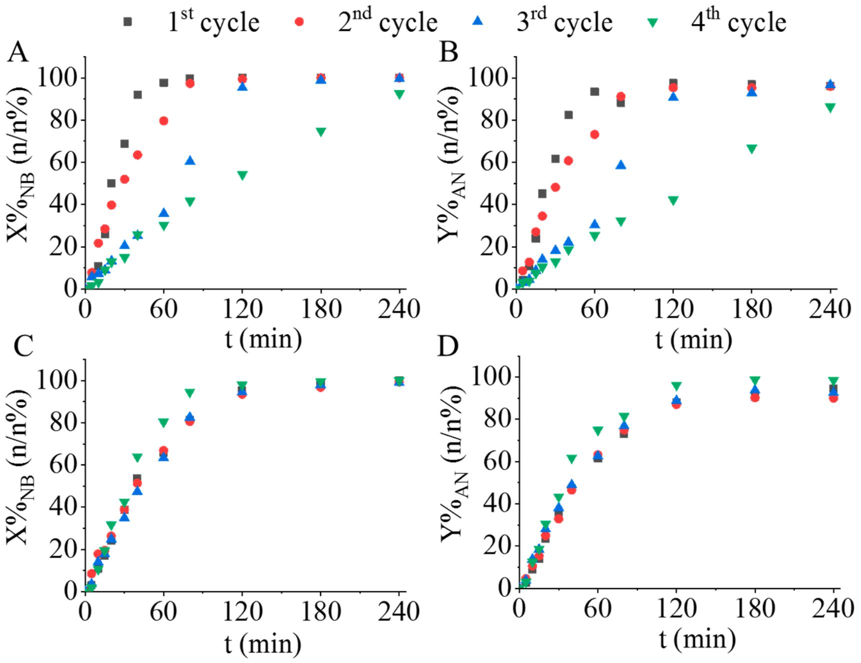

FigS6. Reuse tests of the catalysts. Nitrobenzene conversion and aniline yield vs time of hydrogenation by using Pd/NiFe2O4 (C) and Pd/NiFe2O4-NH2 (D) catalysts.
